# F-Doped In_2_O_3_ Nanofibers for Enhanced BTX Detection with a Portable Wireless Sensing Module

**DOI:** 10.3390/nano16171119

**Published:** 2026-09-06

**Authors:** Jiaqi Yang, Lisen Yuan, Yi Chen, Xiaobin Zhou, Xiaojuan Yan, Gang Zhao, Weiguang Ma

**Affiliations:** 1 College of Physical Electronics Engineering, Shanxi University, Taiyuan 030006, China; yuanlisen@sxu.edu.cn (L.Y.); 202422609002@email.sxu.edu.cn (Y.C.); xbzhou@sxu.edu.cn (X.Z.); yanxj@sxu.edu.cn (X.Y.); 2 State Key Laboratory of Quantum Optics Technologies and Devices, Institute of Laser Spectroscopy, Shanxi University, Taiyuan 030006, China; gangzhao@sxu.edu.cn; 3 China Collaborative Innovation Center of Extreme Optics, Shanxi University, Taiyuan 030006, China

**Keywords:** gas sensor, F-doped In_2_O_3_ nanofiber, BTX detection, machine learning, wireless sensing

## Abstract

Benzene, toluene, and xylene (BTX) gases pose severe threats to public health on account of their toxic, carcinogenic properties and environmental recalcitrance. In addition, their similar molecular structures and chemical inertness complicate the practical quantification of BTX, severely hindering their high-selectivity detection. In this work, a chemiresistive sensor based on F-doped In_2_O_3_ nanofibers was constructed. This strategy successfully enhanced the sensor response values toward benzene, toluene, and xylene by approximately 4.3, 4.5, and 4.2 times, respectively. Meanwhile, the limits of detection were reduced from 1, 0.5, and 0.25 ppm to 0.5, 0.25 and 0.05 ppm, respectively. The theoretical lower detection limits for benzene, toluene, and xylene are as low as 9.9 ppb, 5.7 ppb, and 2.5 ppb, respectively. Machine learning methods were further employed for the identification of BTX gases and their binary mixtures. By capturing the distinctive and reproducible patterns of feature vectors from various analytes, the optimal algorithm established decision boundaries and achieved a classification accuracy of 92.1%. Meanwhile, the reasons for the improved gas sensing performance are discussed. Furthermore, a portable wireless gas sensing module based on F-doped In_2_O_3_ nanofibers was developed, which can be applied for detecting BTX.

## 1. Introduction

Benzene, toluene, and xylene (BTX) are among the most prevalent and hazardous volatile organic compounds (VOCs) in industrial process, coal combustion, and indoor furniture [1,2,3]. Their toxicity, carcinogenicity, and environmental persistence have led to stringent regulatory limits and heightened public health concerns [4,5,6]. Benzene has been classified as a Group 1 carcinogen by the International Agency for Research on Cancer (IARC) [7]. Because of their highly similar molecular structures and their similar chemical structures and low reactivity, BTX species frequently coexist in complex mixtures, making their selective discrimination challenging. Therefore, highly sensitive detection, reliable discrimination, and on-site quantification of BTX remain critical for environmental monitoring, occupational health protection, and air quality assessment.

Conventional analytical techniques, including fluorescence spectroscopy, gas chromatography–mass spectrometry (GC-MS), and photoionization detection (PID), provide high sensitivity and reliable quantitative analysis for BTX detection. However, their widespread application is hindered due to their bulky instrumentation, high cost, complicated operation and prolonged analysis time, making them unsuitable for real-time field detection [8,9,10]. In recent years, novel sensors based on quantum detection mechanisms have become a research hotspot in the field of VOC sensing. Among them, quantum point-contact sensors (QPCSs) are capable of elucidating gas sensing mechanisms through the analysis of energy-level variations, making them particularly suitable for the detection of inert or weakly reactive VOCs [11]. They can achieve selective detection of VOCs in complex gas mixtures at room temperature [12]. Despite their promising performance in laboratory studies, as an emerging sensing technology, QPCSs still require further development in terms of system integration, standardization, and scalability before they can be practically deployed in real-world environmental monitoring applications. In contrast, semiconductor metal oxide (SMO) gas sensors have attracted considerable attention for portable VOC monitoring owing to their low cost, rapid response and compact configuration [13,14]. Among various SMO materials, In_2_O_3_, a n-type semiconductor with wide bandgap, high carrier mobility and excellent chemical stability, has been used for gas sensing applications [15]. Nevertheless, pristine In_2_O_3_ sensors still suffer from relatively high operating temperatures, slow response and recovery process, limited response and poor selectivity, which restrict their practical applications.

To further improve the gas sensing performance of In_2_O_3_, extensive efforts have been devoted through elemental doping, noble metal decoration, morphology engineering, and heterostructure construction. Among these strategies, anion doping has attracted attention owing to its relatively low cost and facile implementation. Compared with cation doping involving isovalent or aliovalent substitutions, anion doping is more effective in tailoring lattice defects, regulating surface chemisorption activity, and modulating the electronic structure of semiconductors, thereby enhancing carrier concentration and surface reaction kinetics [16,17,18]. Owing to its ionic radius comparable to that of O^2−^, F^−^ can be readily incorporated into the oxide lattice to alter surface defect states and electronic structure, leading to enhanced gas sensing performance. This strategy has been reported in SnO_2_ [19], ZnO [20], and TiO_2_ [21] semiconductors. Despite these advances, systematic investigations into F-doped In_2_O_3_ for highly sensitive and selective BTX detection are still unexplored.

In this work, F-doped In_2_O_3_ nanofibers were successfully synthesized via electrospinning followed by high-temperature calcination. The effects of F doping on the microstructure, crystal size, surface defect chemistry, and gas-sensing performance of In_2_O_3_ were systematically investigated. Gas sensing tests demonstrated that F doping significantly enhanced the sensing performance toward BTX gases, with the optimal F doping ratio of 5 at%. At an operating temperature of 240 °C, the optimized sensor exhibited fast response/recovery times of 3/6 s, 2/9 s, and 1/12 s toward 50 ppm benzene, toluene, and xylene, respectively, together with detection limits of 0.5, 0.25 and 0.05 ppm, respectively. To further improve the discrimination capability toward BTX, response features were extracted by principal component analysis (PCA) and combined with a random forest (RF) algorithm, enabling 92.1% identification accuracy for both single BTX gases and binary mixtures. Furthermore, a portable sensing module based on an STM32 microcontroller was developed, achieving real-time quantitative detection of BTX concentrations. Meanwhile, the intrinsic mechanism was further discussed. This work provides a feasible strategy for designing In_2_O_3_-based BTX gas sensors and offers a promising approach for portable BTX monitoring. Given that real-world gas detection is far more complex than laboratory binary component conditions, addressing this challenge is essential for practical sensor applications and will be a key focus of our future work.

## 2. Experimental Section

### 2.1. Preparation of F-Doped In_2_O_3_ Sensing Materials

All chemicals were of analytical grade and used with no further purification, which were provided by Shanghai Aladdin Biochemical Technology Co., Ltd. (Shanghai, China) N,N-dimethylformamide (DMF) was used as the solvent. A 10 mL quantity of DMF was measured out, followed by the sequential addition of 2 mmol In(NO_3_)_3_·4.5H_2_O and stoichiometric amounts of NH_4_F, with the molar ratios of F^−^ to In^3+^ controlled at 0, 3, 5 and 10 at%, respectively. The mixture was stirred at room temperature until complete dissolution. The F-doped In_2_O_3_ nanofibers were synthesized by electrospinning followed by thermal calcination. The precursor solution was transferred into a 10 mL syringe and electrospun using a syringe pump. The feeding rate was maintained at 0.3 mL h^−1^. An applied voltage of 13–18 kV was used, and the distance between the needle tip and collector was fixed at 15–20 cm. The electrospinning process was conducted for 3 h to collect the precursor fibers. The obtained precursor fibers were calcined in air. The temperature was increased from room temperature to 500 °C for 2 h. The resulting products were F-doped In_2_O_3_ nanofibers. According to the F dopant concentrations, the samples were denoted as In_2_O_3_, 3F-In_2_O_3_, 5F-In_2_O_3_, and 10F-In_2_O_3_, corresponding to F^−^/In^3+^ molar ratios of 0, 3, 5, and 10 at%, respectively. Additionally, supplementary data (7 at% F) was also included.

### 2.2. Characterization of In_2_O_3_ and F-In_2_O_3_ Materials

X-ray diffraction (XRD) patterns were identified by a Rigaku D/Max-2550 V X-ray diffractometer (Rigaku International Corporation, Tokyo, Japan) with Cu Kα1 radiation at a wavelength of 1.5406 Å at 40 kV and 200 mA. The morphologies and structures of as-prepared sensing materials were characterized by scanning electron microscopy (SEM, JSM-7500F, JEOL Ltd., Tokyo, Japan) transmission electron microscopy (TEM, EM-3010, 200kV, JEOL Ltd.) and high-resolution transmission electron microscopy (HRTEM, JEOL Ltd.) equipped with energy-dispersive X-ray spectroscopy (EDS, JEOL Ltd.). The elemental composition and valence states of the prepared samples were evaluated by X-ray photoelectron spectroscopy (XPS, ESCALAB 250, ESCALAB 250, Thermo Fisher Scientific, Leicestershire, UK) with an Al Kα radiation source (hν = 1486.7 eV).

### 2.3. Fabrication and Measurement of Gas Sensors

The as-prepared nanofibers with different F doping levels were finely ground and dispersed in ethanol to form homogeneous slurries. The slurries were coated onto planar side-heated electrode substrates. After drying, the electrode chips were calcined at 400 °C for 2 h in a muffle furnace to enhance the adhesion and crystallinity of the sensing layers. Subsequently, the obtained chips were welded onto four-cornered base and further aged at 180 °C for 7 days. Accordingly, four types of gas sensors, including In_2_O_3_, 3F-In_2_O_3_, 5F-In_2_O_3_, 7F-In_2_O_3_ and 10F-In_2_O_3_, were fabricated. For each doping concentration, we prepared two sensors to evaluate the stability.

The gas-sensing properties of the sensors were tested using a static system in this experiment, where two 1 L gas cylinders were employed to simulate the air atmosphere and the target gas atmosphere, respectively. A certain concentration of liquid was injected into the target gas cylinder using a microsyringe. The needed concentration of the tested VOC gases can be calculated using static liquid gas distribution Equation (1):

(1) $$C_{1}=\frac{24.1\times \omega \times \rho \times V_{1}}{{M\times V}_{2}}$$

where $C_{1}$ (ppm) is the target gas concentration, ω (wt%) is the mass percentage of the liquid, ρ (g/mL) is the density of the liquid, V_1_ (mL) is the volume of liquid, V_2_ (L) is the volume of the chamber and M (g/mol) is the molecular weight of the target gas. In this work, a Fluke 8846A high-precision digital multimeter (Fluke Corporation, Everett, WA, USA) coupled with a computer was used to record the dynamic resistance changes of the sensors in ambient air (R_a_) and target gas (R_g_) atmospheres at different operating temperatures. In order to discriminate the BTX, in this work, the gas response value is defined as follows: $S\;=\frac{\left|R_{a}-R_{g}\right|}{R_{g}}\times 100\%$. The response time and recovery time can be calculated by the resistance change of |R_g_ − R_a_| × 90%, respectively.

## 3. Results and Discussion

### 3.1. Morphology Characterization

**Figure 1:**
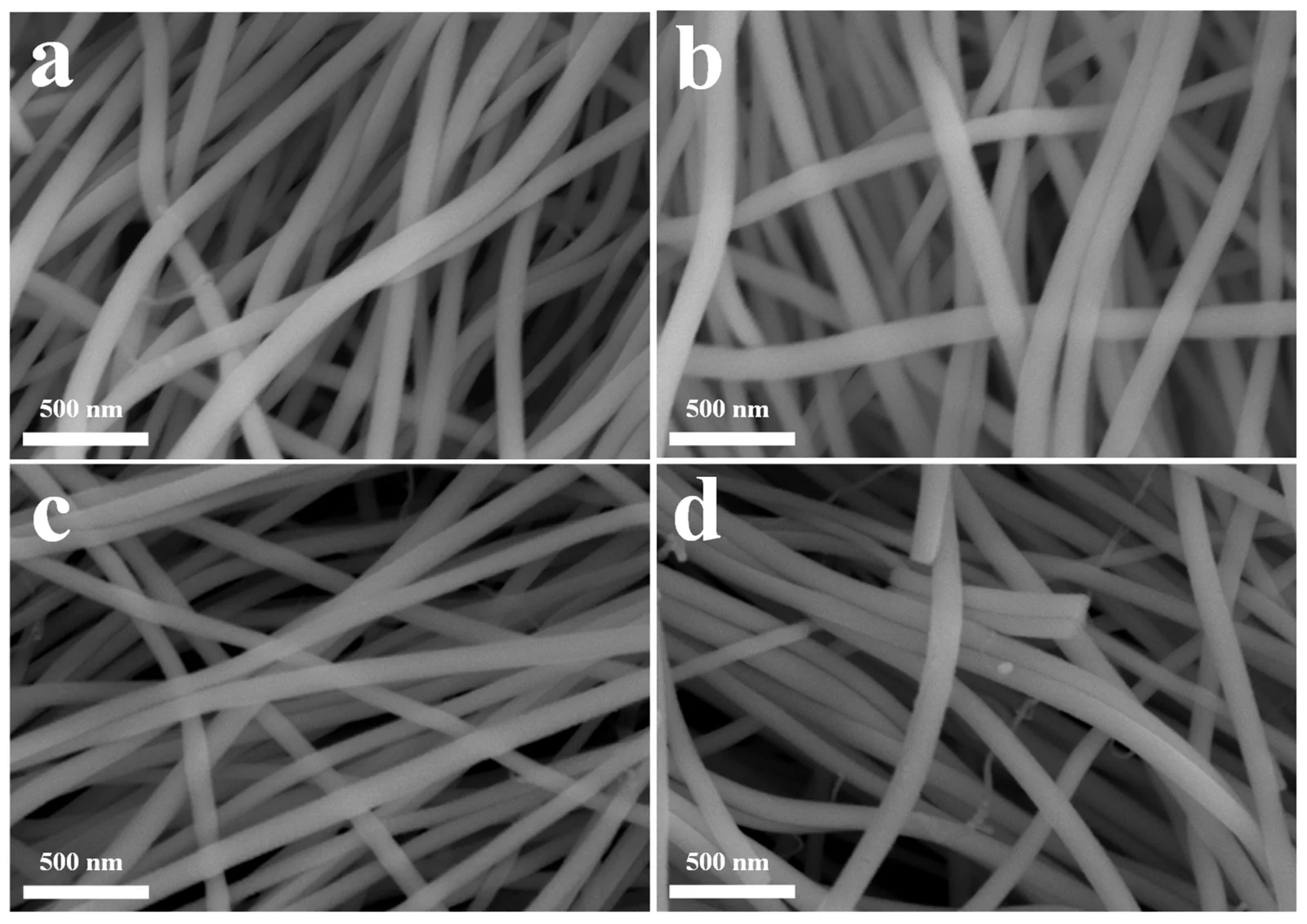
SEM images of (**a**) In_2_O_3_, (**b**) 3F-In_2_O_3_, (**c**) 5F-In_2_O_3_ and (**d**) 10F-In_2_O_3_ nanofibers.

As shown in Figure 1a, In_2_O_3_ possesses a 1D nanofiber structure with a smooth surface. SEM images of In_2_O_3_ with varying F-doped levels are presented in Figure 1b–d. No remarkable morphological differences are observed between F-In_2_O_3_ and pristine In_2_O_3_ samples, indicating that F doping has a negligible effect on the nanofiber morphology.

**Figure 2:**
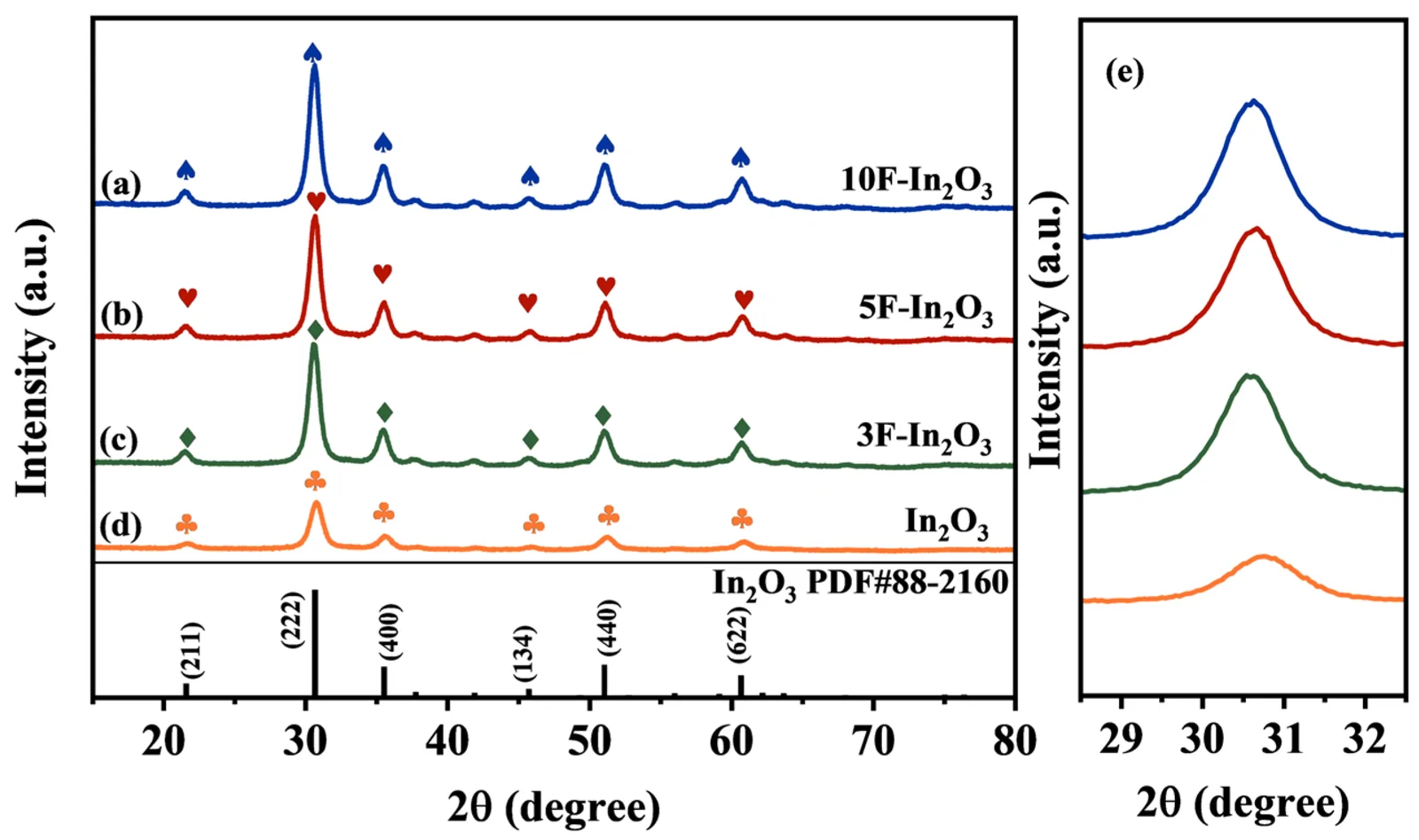
X-ray diffraction patterns of (**a**) In_2_O_3_, (**b**) 3F-In_2_O_3_, (**c**) 5F-In_2_O_3_, (**d**) 10F-In_2_O_3_, and (**e**) comparison of In_2_O_3_ (222) peaks.

The crystal structures of the samples were characterized by X-ray diffraction (XRD), and the results are shown in Figure 2a–d. All diffraction peaks of the samples correspond to the standard card of indium oxide (PDF#88-2160), demonstrating that F doping does not alter the cubic phase structure of In_2_O_3_. The crystallite sizes of In_2_O_3_, 3F-In_2_O_3_, 5F-In_2_O_3_, and 10F-In_2_O_3_, calculated using the Scherrer equation, were found to be 7.24 nm, 8.42 nm, 8.83 nm, and 8.61 nm, respectively. With increasing F content, the crystallite size of the samples first increases and then decreases. The 5F-In_2_O_3_ exhibits the largest grain size. This non-monotonic trend is consistent with the grain growth behavior observed in other doped In_2_O_3_ systems [22]. At low F-doping levels (0–5 at%), F ions preferentially substitute for lattice O^2−^ sites. The oxygen vacancies generated by this aliovalent substitution significantly accelerate ion diffusion in the lattice and promote grain growth, a phenomenon also observed in other oxide ceramics where oxygen vacancies promote diffusion-controlled coarsening [23]. As the F content increases beyond the solid solubility limit (10 at%), partial F ions are likely to occupy interstitial sites or segregate at grain boundaries, leading to enhanced lattice distortion. Such grain boundary segregation can impede grain boundary migration and suppress crystallite growth, resulting in a slight decrease in crystallite size [24,25]. Because the ionic radius of F^−^ (0.133 nm) is slightly smaller than that of O^2−^ (0.140 nm), substitutional F^−^ doping would contract the lattice. When F^−^ ions substitute for lattice O^2−^ ions, the reduced ionic radius would be expected to decrease the lattice spacing, resulting in a shift in the diffraction peaks toward higher 2θ angles according to Bragg’s law. However, the enlarged view of the (222) diffraction peak reveals a slight shift toward lower 2θ angles, opposite to the expected trend. This phenomenon may be attributed to the formation of oxygen vacancies, lattice distortion and tensile strain, which collectively lead to lattice expansion despite the smaller ionic radius of F^−^ [25,26] In F-doped SnO_2_ systems, Wang et al. [19] reported that the ionic radius mismatch between F^−^ and O^2-^ induced lattice distortion and promoted the formation of oxygen vacancies. This structural evolution is consistently supported by subsequent XPS O 1s analysis.

To distinguish surface adsorption or secondary fluoride formation from lattice doping, the crystallite size variation obtained from XRD measurements are further analyzed. According to the classical grain boundary pinning theory, both solute atoms segregated at grain boundaries (corresponding to surface adsorption) and second-phase particles (corresponding to secondary fluorides) exert a unidirectional inhibitory effect on grain growth, leading to a monotonic decrease in grain size with increasing impurity concentration. However, the crystallite sizes of In_2_O_3_, 3F-In_2_O_3_, 5F-In_2_O_3_, and 10F-In_2_O_3_, calculated using the Scherrer equation, are to be 7.24 nm, 8.42 nm, 8.83 nm, and 8.61 nm, respectively. The crystallite sizes of our work exhibit a non-monotonic trend of first increasing and then decreasing with F doping, and notably, even for the 10F-In_2_O_3_ sample, the grain size remains larger than that of undoped In_2_O_3_. This is inconsistent with the predictions of the solute drag or Zener pinning mechanisms, thereby ruling out the possibilities of surface adsorption or secondary fluoride formation [27]. The result further confirms that F is incorporated into the In_2_O_3_ lattice.

**Figure 3:**
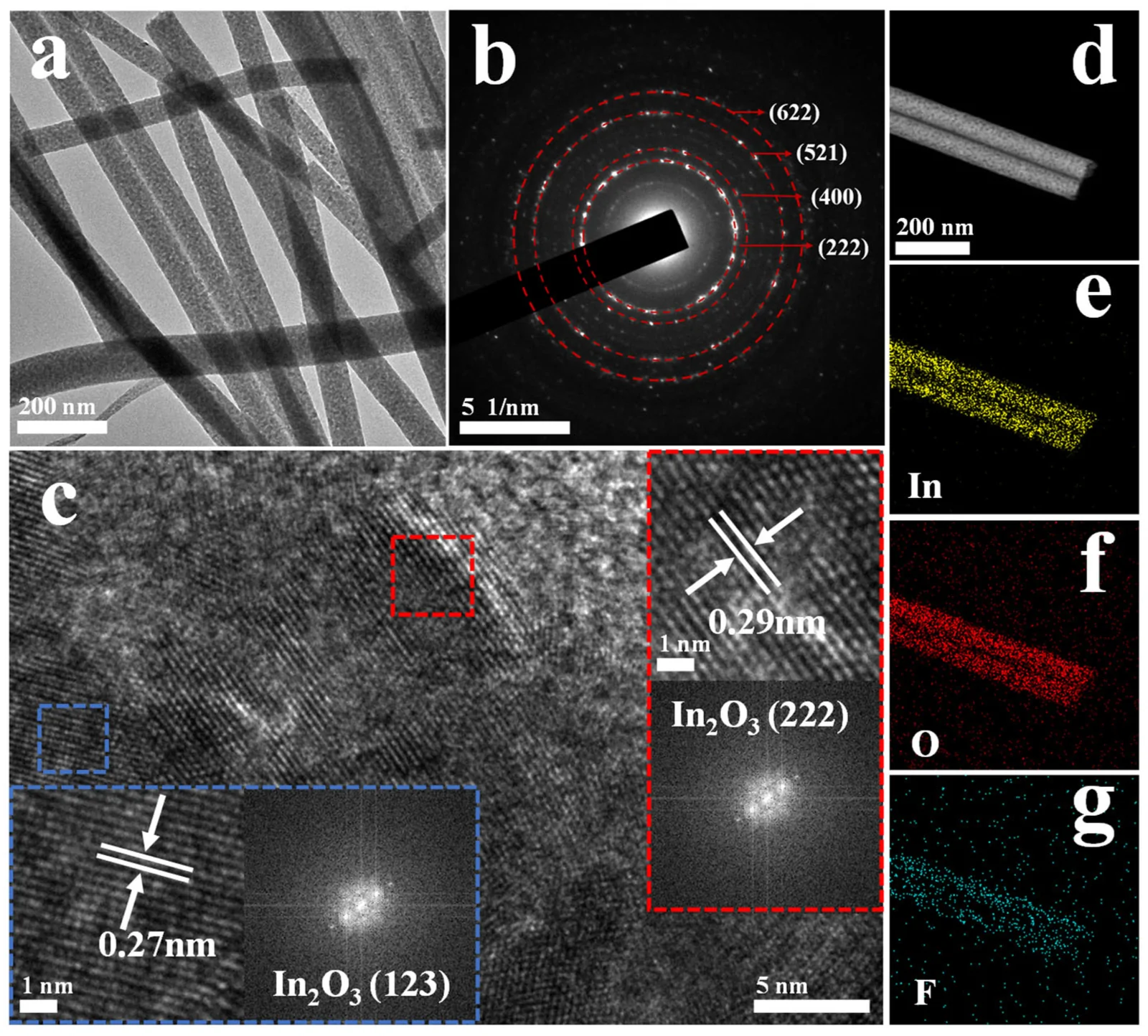
(**a**) TEM image of 5F-In_2_O_3_; (**b**) SAED image; (**c**) HRTEM image, with magnified views of the red and blue regions showing high-resolution lattice fringes and the corresponding FFT patterns; (**d**) STEM image; (**e**–**g**) EDS elemental mapping images of In, O, and F.

TEM image in Figure 3a reveals that 5F-In_2_O_3_ nanofibers possess uniform morphology with dense and porous interior structures. The SAED pattern of 5F-In_2_O_3_ displays clear diffraction rings corresponding to the (222), (400), (521), and (622) planes of cubic In_2_O_3_, indicating a polycrystalline structure (Figure 3b). HRTEM images exhibit clear crystalline lines for the 5F-In_2_O_3_ sample, suggesting their crystalline nature. The lattice fringes with interplanar spacings of 0.29 nm and 0.27 nm can be clearly observed in Figure 3c, which correspond to the (222) and (123) crystal planes of cubic In_2_O_3_ (PDF#88-2160), respectively. As shown in Figure 3d–g, the EDS elemental mapping results show that In, O and F elements are uniformly distributed throughout the 5F-In_2_O_3_ nanofiber. Combined with the above morphological evidence and XRD characterization results, we can confirm that F is successfully doped into In_2_O_3_.

**Figure 4:**
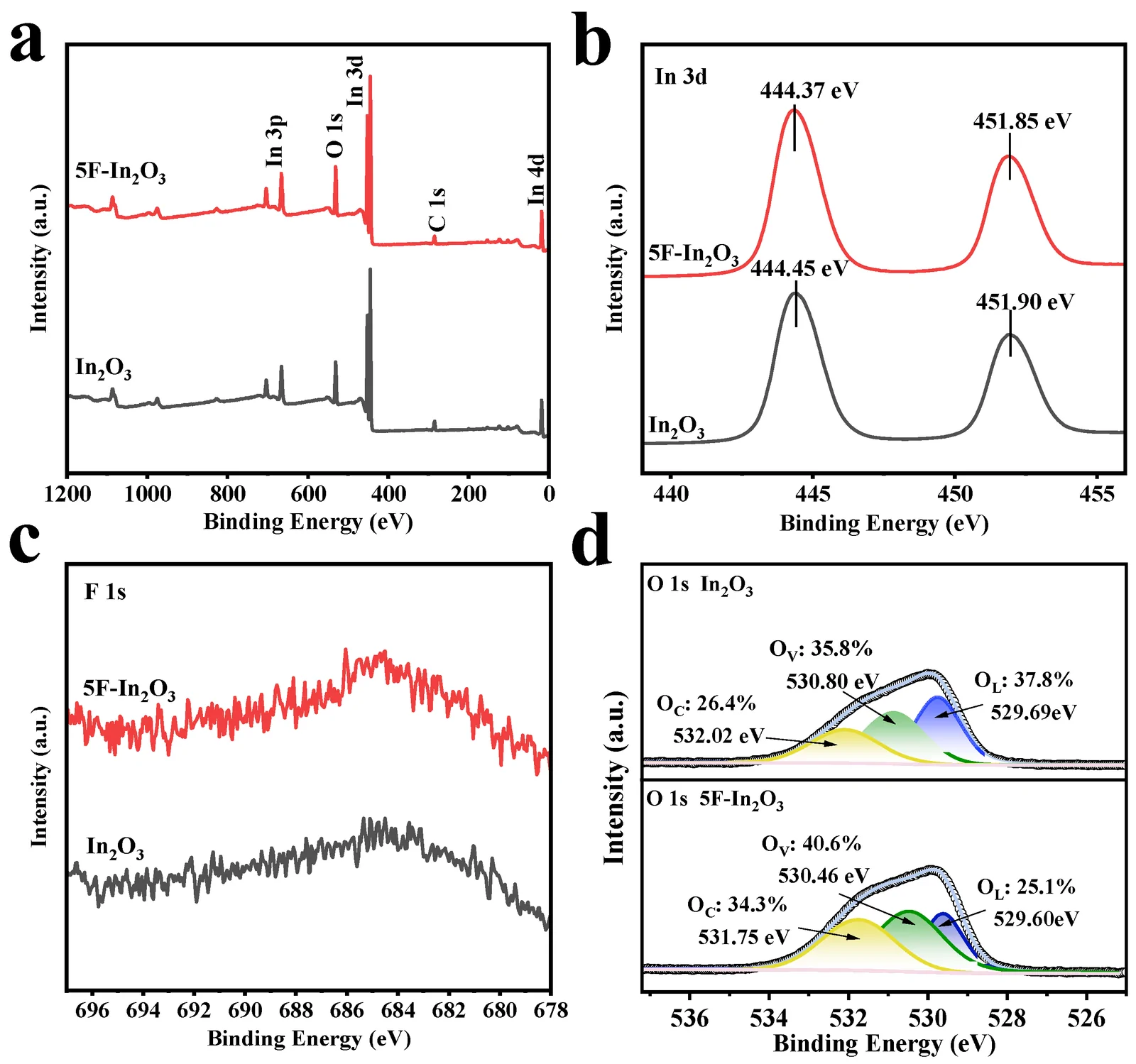
(**a**) XPS survey of In_2_O_3_ and 5F-In_2_O_3_, XPS core levels of (**b**) In 3d, (**c**) F 1s, and (**d**) O1s fitted spectra.

XPS was used to analyze the chemical valence states. Figure 4a shows the survey spectra of In_2_O_3_ and 5F-In_2_O_3_ nanofibers. Peaks of In, O, and C elements are clearly detected, whereas F signal is not observed due to its low doping concentration. In the In 3d spectrum of Figure 4b, the peaks located at 451.90 eV (In 3d_3/2_) and 444.45 eV (In 3d_5/2_) confirm that In ions exist in the +3 valence. Meanwhile, it can be observed that both the In 3d_3/2_ and In 3d_5/2_ peaks of the 5F-In_2_O_3_ sample have a slight shift to lower binding energies, which is attributed to the introduction of oxygen vacancies by F doping and the change in the electron cloud density around the In species [20]. The high-resolution O 1s XPS spectra of In_2_O_3_ and 5F-In_2_O_3_ are displayed in Figure 4d, respectively. Both spectra are fitted with three Gaussian peaks: the peak near 529.6 eV is assigned to lattice oxygen (O_L_), the peak at 530.5–530.8 eV to oxygen vacancies (O_V_), and the peak at 531.7–532.0 eV to surface-chemisorbed oxygen (O_C_). After F doping, the O_V_ proportion rises from 35.8% to 40.6%, and the O_C_ proportion increases from 26.4% to 34.3%, while the O_L_ fraction drops from 37.8% to 25.1%. The higher O_V_ concentration provides more active sites and promotes the generation of chemisorbed oxygen species (O_2_^−^, O^−^ and O^2−^); the O_C_ species can directly oxidize the target gas and induce a pronounced resistance change, which can promote sensing performance [28,29].

### 3.2. Gas Sensing Performance Investigation

**Figure 5:**
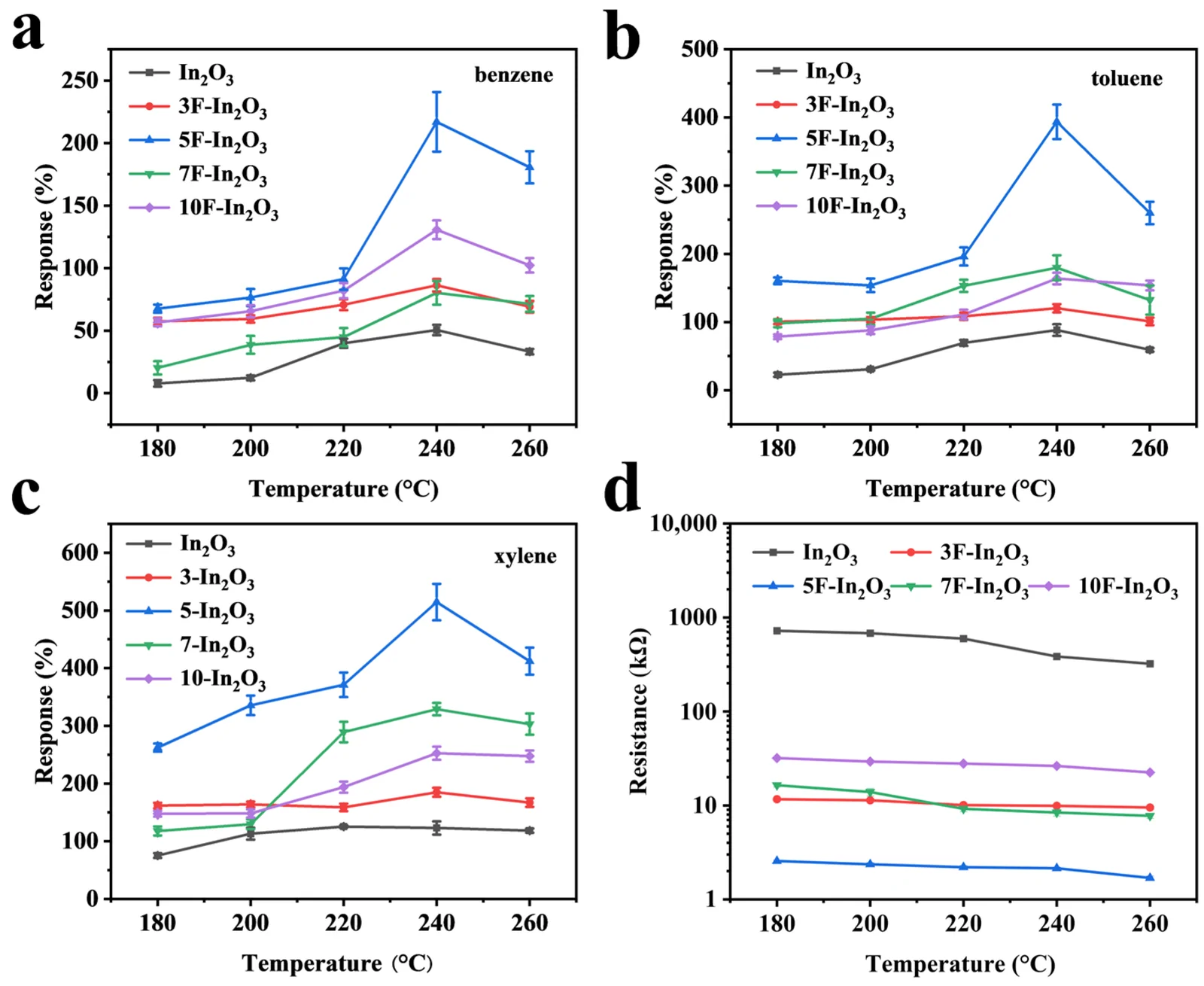
Gas responses of In_2_O_3_, 3F-In_2_O_3_, 5F-In_2_O_3_,7F-In_2_O_3_ and 10F-In_2_O_3_ sensors to 50 ppm BTX at different operating temperatures: (**a**) benzene, (**b**) toluene, (**c**) xylene; (**d**) baseline resistance of all sensors at different operating temperatures.

To evaluate the sensing performance, In_2_O_3_ and F-In_2_O_3_ nanofiber sensors were fabricated. Figure 5a–c display the gas responses of fabricated sensors toward 50 ppm BTX that were tested at the range of 180 °C to 260 °C. The response shows a volcano-shaped variation as the operating temperature rises. As shown in Figure 5a, the F-In_2_O_3_ sensors exhibit higher responses to benzene than the In_2_O_3_ sensor. At an operating temperature of 240 °C, the 5F-In_2_O_3_ sensor shows the highest response to 50 ppm benzene, with a value of 217, which is approximately 4.3 times that of the In_2_O_3_ sensor. From Figure 5b, it can be seen that the 5F-In_2_O_3_ sensor exhibits the highest response to 50 ppm toluene at 240 °C, which is about 4.5 times higher than that of the In_2_O_3_ sensor. Figure 5c presents the response of the sensors to xylene; the 5F-In_2_O_3_ sensor also achieves the maximum response to 50 ppm xylene at 240 °C, which is approximately 4.2 times that of the In_2_O_3_ sensor. Compared with the In_2_O_3_ sensor, the responses of 5F-In_2_O_3_ sensors to BTX gases are significantly improved at the optimal working temperature of 240 °C. The gas response of all sensors follows the order xylene > toluene > benzene, which can be rationalized by the distinct molecular structures of benzene, toluene and xylene. Specifically, xylene, toluene and benzene possess two, one and zero methyl substituents, respectively. Greater methyl substitution strengthens molecular adsorption onto the materials surface, giving rise to an elevated gas response toward xylene [30]. The 5F-In_2_O_3_ sensor affords the highest sensing response at its optimal operating temperature. Excessive F dopant instead diminishes the response, suggesting the necessity of tuning doping content to an optimal level. In addition, the variation in baseline resistance (Rₐ) with temperature was also investigated. Figure 5d presents the change in baseline resistance of the sensors over the temperature range of 180 °C to 260 °C. As can be seen, the baseline resistance decreases with increasing operating temperature, which is consistent with previous reports [31]. Meanwhile, it is also noted that as the F doping amount increases, the baseline resistance first decreases and then increases. This phenomenon may be related to the existing form of F ions, which will be discussed in relation to the gas sensing mechanisms.

**Figure 6:**
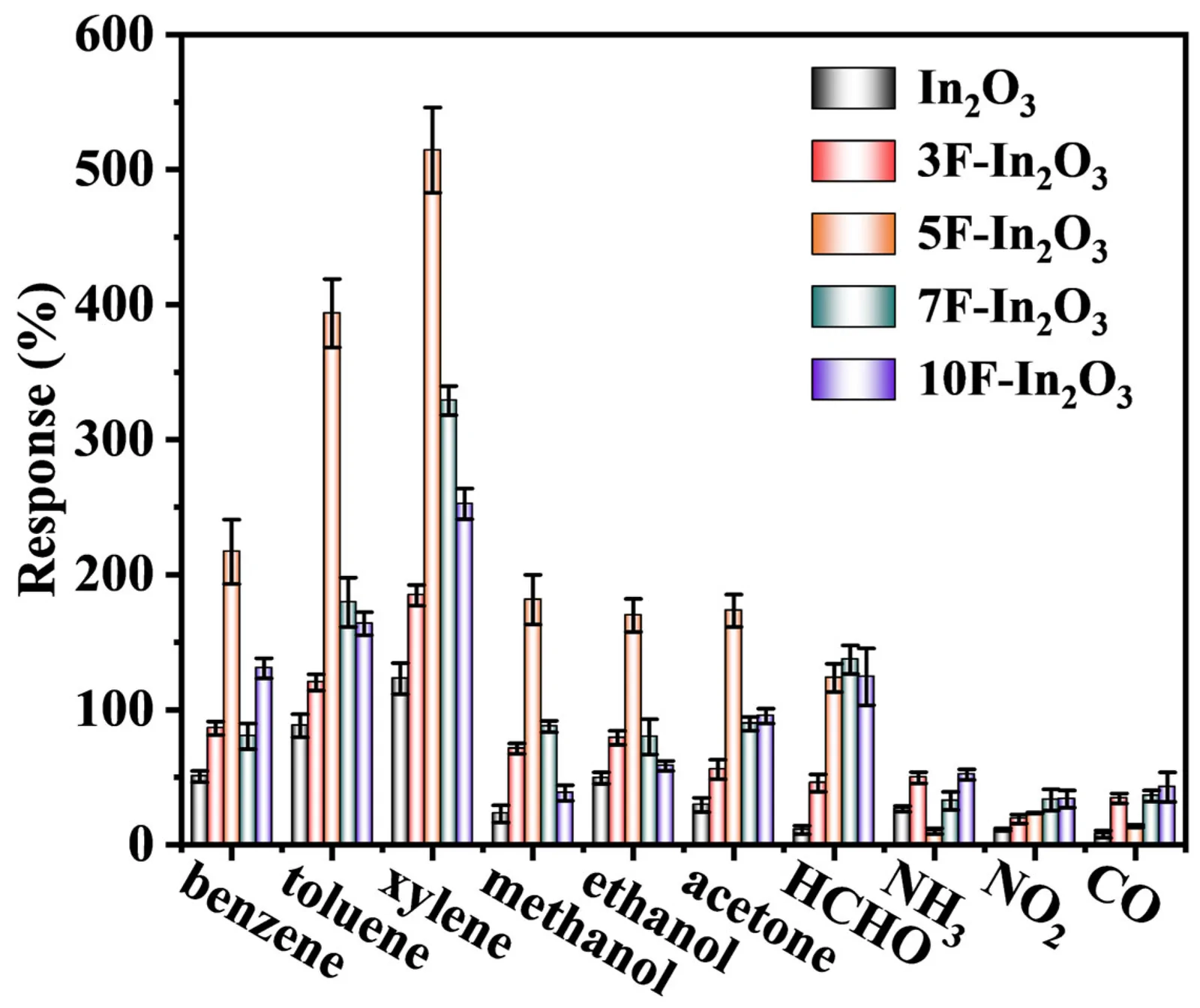
Gas responses of In_2_O_3_, 3F-In_2_O_3_, 5F-In_2_O_3_, 7F-In_2_O_3_ and 10F-In_2_O_3_ sensors to various gases at 240 °C.

Figure 6 compares the response differences of In_2_O_3_, 3F-In_2_O_3_, 5F-In_2_O_3_, and 10F-In_2_O_3_ sensors to 50 ppm BTX (benzene, toluene, xylene), and other interfering gases (methanol, ethanol, acetone, formaldehyde, NH_3_, NO_2_, CO) at 240 °C. Among them, the 5F-In_2_O_3_ sensor exhibits the best overall BTX sensing performance. Additionally, supplementary data (7 at% F) was added to confirm the truly optimal ratio in Figure 5 and Figure 6. Although the 7F-In_2_O_3_ sensor exhibits enhanced selectivity to BTX compared with undoped In_2_O_3_, the gas responses of the 7F-In_2_O_3_ sensor are lower than that of the 5F-In_2_O_3_ sensor at the same concentration. The discrimination of BTX gases will be analyzed in the subsequent discussion.

**Figure 7:**
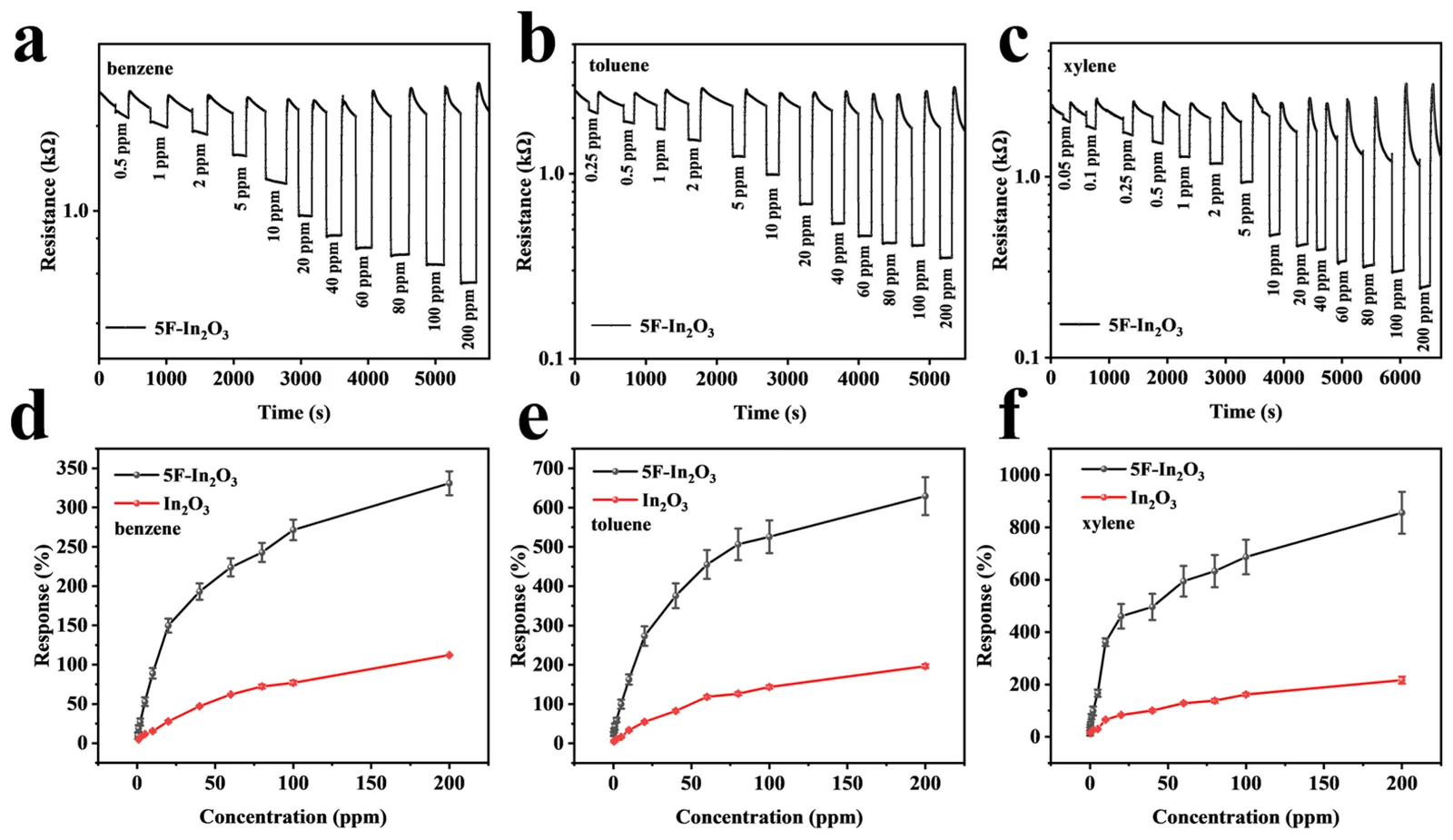
Dynamic responses of 5F-In_2_O_3_ sensor to various BTX concentrations at 240 °C: (**a**) benzene, (**b**) toluene, (**c**) xylene. Corresponding response–concentration relationship curves of (**d**) benzene, (**e**) toluene, (**f**) xylene.

The responses of the fabricated sensors toward benzene, toluene, and xylene show a monotonic increase with gas concentration. This behavior agrees well with the typical sensing behavior of n-type MOS materials. Given the hazardous nature of BTX gases, the gas sensing tests were further extended to lower concentration levels. As shown in Figure 7a–c, the 5F-In_2_O_3_ sensor exhibits response values of approximately 12, 24, and 21 toward 500 ppb benzene, 250 ppb toluene, and 50 ppb xylene, respectively, indicating a detection limit well below occupational exposure limits [32,33,34,35]. In contrast, the In_2_O_3_ sensor only achieves responses of 5, 7 and 15 to 1 ppm benzene, 500 ppb toluene and 250 ppb xylene, respectively. It reveals that F doping further decreases the limit of detection of the In_2_O_3_ sensor. Figure 7d–f clearly illustrate the linear correlation between gas response and BTX concentration for the In_2_O_3_ and 5F-In_2_O_3_ sensors. At low BTX concentrations, sensor response rises rapidly and follows a linear trend. With further increasing BTX concentrations, the growth rate of response value becomes slow and present an obvious saturation effect. This behavior can be understood by surface adsorption process. At low analyte levels, only partial BTX molecules interact with surface adsorbed oxygen species, leading weak sensing signals. When BTX concentration increases, abundant target molecules engage in redox reactions with surface oxygen species, which significantly boosts the sensor response. At high BTX concentrations, surface active adsorption sites are occupied by BTX molecules. The shortage of available adsorbed oxygen restricts further redox reactions and sensing enhancement [36,37]. Furthermore, the 5F-In_2_O_3_ sensor displays a steeper slope under low BTX concentrations compared with the In_2_O_3_ sensor. The linear regression analysis (Figures S3–S5) yields well-defined calibration curves for benzene, toluene, and xylene over the concentration ranges of 0.5–10 ppm, 0.25–10 ppm, and 0.05–10 ppm, respectively. The corresponding fitting equations are y = 8.3x + 8.7 (R^2^ = 0.985), y = 14.3x + 25.9 (R^2^ = 0.981), and y = 32.7x + 26.3 (R^2^ = 0.980), all demonstrating excellent linearity. By incorporating the normalized baseline resistance in air (Table S1), the theoretical detection limits of the 5F-In_2_O_3_ sensor for benzene, toluene, and xylene are determined to be 9.9 ppb, 5.7ppb, and 2.5 ppb, respectively. This confirms its superior sensitivity, which endows the F-In_2_O_3_ sensor with great potential for trace BTX detection.

**Figure 8:**
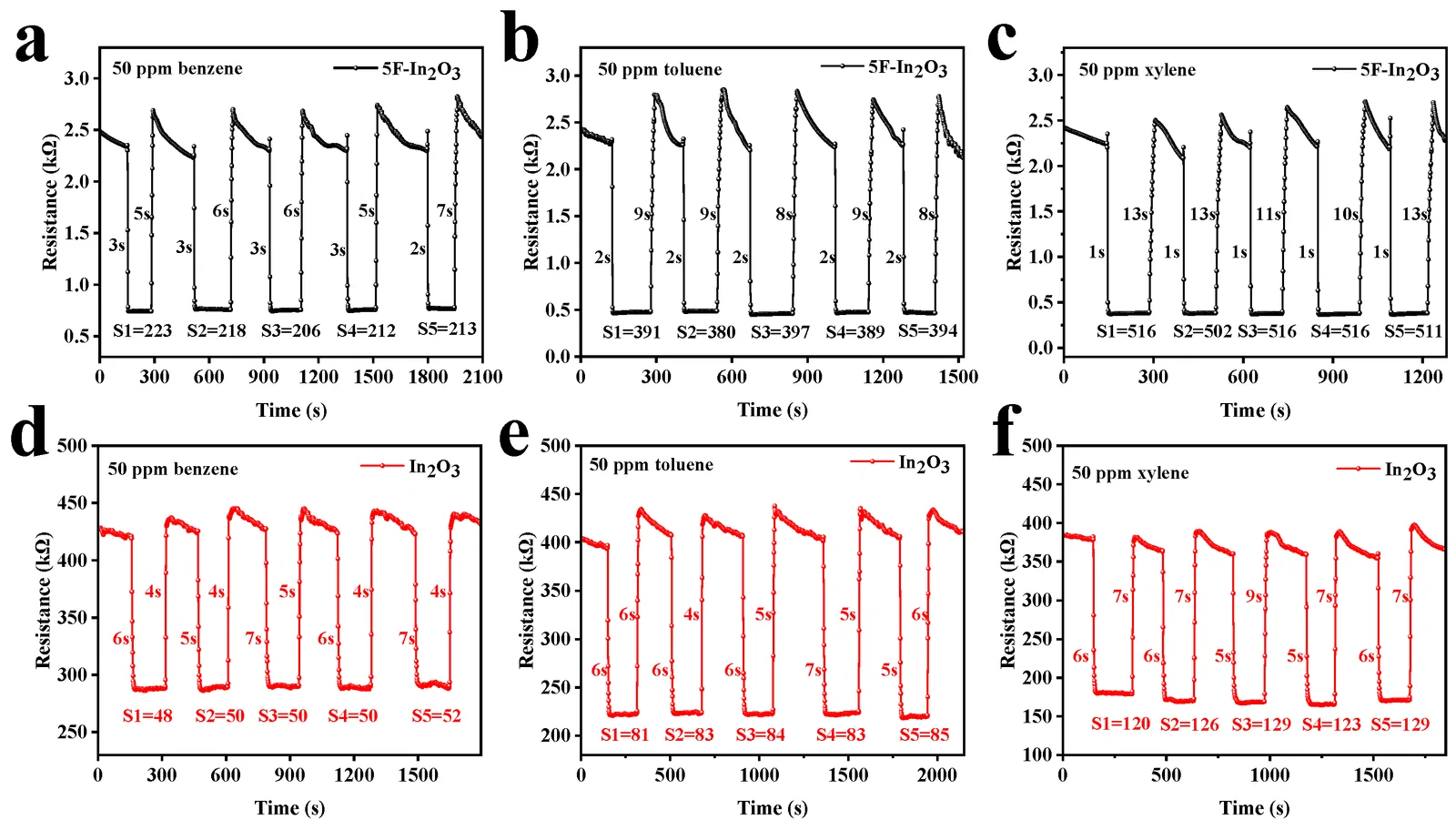
Transient curves of 5F-In_2_O_3_ (**a**–**c**) and In_2_O_3_ (**d**–**f**) sensors to 50 ppm benzene, toluene, and xylene at 240 °C.

Figure 8a–f presents the transient cycle curves of the 5F-In_2_O_3_ and In_2_O_3_ sensors to 50 ppm benzene, toluene, and xylene at 240 °C. Upon exposure to the BTX gases, the resistance of both sensors decreases rapidly. The response values of the In_2_O_3_ sensor to 50 ppm benzene, toluene, and xylene are about 50, 83, and 125, respectively. The average response times of the In_2_O_3_ sensor to benzene, toluene, and xylene are 6 s, 6 s, and 6 s, respectively, whereas those of the 5F-In_2_O_3_ sensor are shortened to 3 s, 2 s, and 1 s, indicating that F species can accelerate the surface reaction kinetics of BTX molecules. The average recovery times of the In_2_O_3_ sensor to benzene, toluene, and xylene were 4 s, 5 s, and 7 s, respectively, whereas those of the 5F-In_2_O_3_ sensor were slightly prolonged to 6 s, 9 s, and 12 s. This behavior may be attributed to the stronger adsorption of BTX molecules and their oxidation products on the materials surface. As reported in DFT studies, enhanced adsorption energy leads to significantly prolonged recovery times [38]. The response values of the 5F-In_2_O_3_ sensor to 50 ppm benzene, toluene, and xylene are about 214, 390, and 512, revealing the excellent repeatability. The reproducibility was evaluated using two independent sensors. The average responses of the optimal 5F-In_2_O_3_ sensor to 50 ppm benzene, toluene, and xylene were determined to be 217.0 ± 23.8, 393.7 ± 25.3, and 514.5 ± 31.6 (SD, n = 2), with corresponding relative standard deviations (RSD) of 11.0%, 6.4%, and 6.1%, respectively.

Meanwhile, long-term stability was further evaluated. As shown in Figure S2a, the 5F-In_2_O_3_ sensor maintains stable gas sensing performance over a 15-day period. The response values toward BTX gases show no significant degradation, confirming its good stability and reproducibility. Meanwhile, Figure S2b presents the response values of the 5F-In_2_O_3_ sensor to 50 ppm BTX under different relative humidity (RH) conditions. As the relative humidity increased from 30% to 90%, the responses of the sensor toward 50 ppm BTX showed a gradual decrease. At 30% RH, the response values for benzene, toluene, and xylene were 217.0, 393.7, and 514.5, respectively, which decreased to 105.5, 144.6, and 190.6, respectively, when the RH was elevated to 90%. It is evident that the response attenuation became more pronounced with increasing RH. This phenomenon can be attributed to the competitive adsorption between water molecules and BTX molecules on the active sites of the material surface [39]. Water molecules can occupy part of the oxygen vacancies and adsorption sites, thereby limiting the adsorption and subsequent oxidation of BTX molecules, which ultimately results in reduced sensor responses. Additionally, the 5F-In_2_O_3_ sensor shows fast response and recovery behavior and a low detection limit toward BTX gases, which are superior to those of previous metal oxide gas sensors (Table 1).

To enable accurate discrimination of BTX gases and their binary mixtures, machine learning models were employed. The dynamic sensing features, including response value (S), response time (t_1_), and recovery time (t_2_), were extracted from the response profiles. After Z-score normalization, the processed features were used for model training and evaluation. PCA was further applied to visualize the feature distribution. Eight classification models, including Random Forest (RF), Extreme Gradient Boosting (XGBoost), Support Vector Machine (SVM), Gradient Boosting Decision Tree (GBDT), K-Nearest Neighbors (KNN), Multilayer Perceptron (MLP), Logistic Regression (LR), and Convolutional Neural Network (CNN), were compared. Their performances were evaluated using accuracy, five-fold cross-validation, confusion matrices, ROC curves, and learning curves.

The dataset consists of six categories of gas samples. Each pure-gas category (benzene B, toluene T, xylene X) contains three samples; each binary mixed-gas category (B + T, B + X, T + X) contains nine samples with three mixing ratios of 1:1, 1:2 and 2:1, and three parallel samples are prepared for each ratio. The original dataset totals 36 samples. To address the class imbalance issue and mitigate overfitting risks caused by limited samples, a Gaussian noise augmentation strategy is adopted in this work. Each category is augmented to 25 samples, constructing a balanced dataset with a total of 150 samples. The gas concentration was set to 50 ppm in experimental tests. The dataset was randomly split into a training set (75%) and a test set (25%).

Considering the limited size of the dataset (150 samples after data augmentation), all hyperparameters were manually preset based on prior experience and small-sample modeling criteria. To objectively evaluate the generalization performance of each model, a hierarchical evaluation framework of “internal validation plus independent testing” is established in this study. Five-fold cross-validation was performed within the training set to preliminarily assess model stability. To prevent excessive exploitation of validation information, cross-validation results are not used for final model selection. Meanwhile, 25% of the samples are reserved as an independent test set that is completely excluded from training. The final evaluation was carried out after all model training, hyperparameter fixation and cross-validation were completed, and the obtained results served as the final metric for measuring model generalization capability.

To prevent overfitting and data leakage, preventive measures were implemented from both data processing and model training perspectives. The statistical parameters for data augmentation were derived solely from original samples, and the generated samples were clipped to a globally reasonable range, ensuring that the augmentation process did not rely on the distribution of the test set. The transformation parameters for standardization and feature selection were strictly fitted only on the training set, and stratified sampling was adopted to maintain consistent class proportions, fundamentally blocking the paths of data leakage. At the model level, all hyperparameters were manually preset based on the training set and internal validation set, and intervention of the test set in parameter tuning was strictly prohibited. Meanwhile, an early stopping mechanism and strong regularization worked synergistically to suppress excessive iteration and weight inflation, effectively mitigating the risk of overfitting with limited samples, and guaranteeing the objectivity and reliability of the final generalization performance measurement.

**Figure 9:**
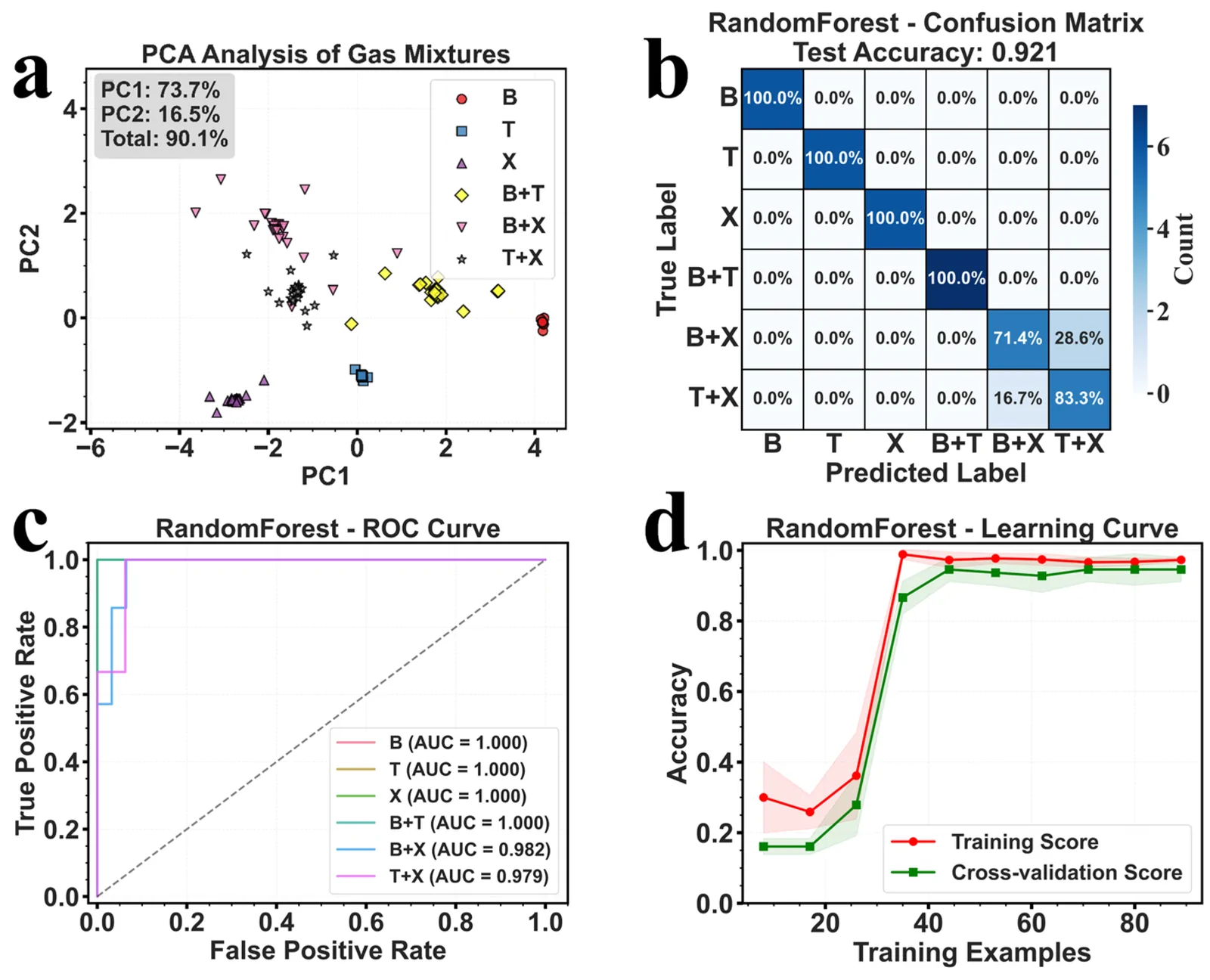
Performance evaluation of RF model for classifying BTX gases and their binary mixtures. (**a**) PCA projection of the BTX dataset, (**b**) confusion matrix of the RF model on the test set, (**c**) ROC curves and AUC values of RF model, (**d**) learning curves of RF model.

Figure 9a shows the PCA projection of the BTX sensing dataset. PC1 and PC2 account for 73.7% and 16.5% of the total variance, respectively, resulting in a cumulative explained variance of 90.1%. This result indicates that the two-dimensional feature space retains most of information from the original dataset. The three BTX gases form distinct clusters, demonstrating their different sensing characteristics. The binary mixtures are located between the corresponding single gas clusters, which agrees with the combined contribution of the individual components to the gas response. Notably, considerable overlap is observed between the benzene–xylene and toluene–xylene clusters, suggesting high feature similarity and potential difficulty in their discrimination. The classification performance of the eight models is summarized in Table 2. Among these models, RF achieved the best overall performance, with a test accuracy of 92.1%. It was the highest among these evaluated models. The superior performance of RF demonstrates the capability for distinguishing similar BTX gases and their mixtures. Further evaluation of RF was conducted using confusion matrices, ROC curves, and learning curves. As shown in Figure 9b, benzene, toluene, xylene, and the benzene–toluene mixture can be correctly identified with 100% accuracy. The misclassifications mainly occurred between benzene–xylene and toluene–xylene mixtures, consistent with the overlapping regions observed in the PCA plot. The ROC curves in Figure 9c show AUC values of 1.000 for benzene, toluene, xylene, and benzene–toluene mixtures. The AUC values for the benzene–xylene and toluene–xylene mixtures are 0.982 and 0.979, respectively, indicating excellent classification capability. The learning curve of RF is presented in Figure 9d. Both training and cross-validation scores increased with the training set size and gradually reached stable values. The small gap between the two curves indicates good generalization ability without obvious overfitting or underfitting. These results confirm the robustness of RF for BTX classification and highlight the potential for reliable identification of BTX gases and their binary mixtures.

### 3.3. Practical Application Evaluation and Mechanism Analysis

#### 3.3.1. Mechanism Analysis

As an n-type semiconductor, In_2_O_3_ exhibits gas sensing properties that are governed by the modulation of the electron depletion layer (EDL). Variations in the EDL directly alter the surface resistance of sensing materials. In this work, the relevant surface reactions are proposed as follows:

O_2_ (ads) + e^−^→O_2_^−^ (ads)  (<100 °C) (2)

(3) $$O_{2}\;(\mathrm{ads})+e^{-}\to O^{-}\;(\mathrm{ads})\;\;(100\;{}^{\circ}C\leq T\leq 300\;{}^{\circ}C)$$

O_2_ (ads) + e^−^→O^2−^ (ads)  (>300 °C) (4)

(5) $$C_{6}H_{6}\;(\mathrm{ads})+15O^{-}\to 3H_{2}O+6{\mathrm{CO}}_{2}+15e^{-}(\mathrm{ads})\;\;(100\;{}^{\circ}C\leq T\leq 300\;{}^{\circ}C)$$

(6) $$C_{7}H_{8}\;(\mathrm{ads})+18O^{-}\to 4H_{2}O+7{\mathrm{CO}}_{2}+18e^{-}(\mathrm{ads})\;\;(100\;{}^{\circ}C\leq T\leq 300\;{}^{\circ}C)$$

(7) $$C_{8}H_{10}\;(\mathrm{ads})+21O^{-}\to 5H_{2}O+8{\mathrm{CO}}_{2}+21e^{-}(\mathrm{ads})\;\;(100\;{}^{\circ}C\leq T\leq 300\;{}^{\circ}C)$$

According to Equations (2)–(4), the operating temperature plays a critical role in regulating the surface oxygen species of gas sensors [48]. At the optimized operating temperature of 240 °C, O^−^ species are the predominant reactive oxygen species on the surface, which subsequently react with BTX molecules through the oxidation processes described in Equations (5)–(7). The superior sensing performance of the 5F-In_2_O_3_ sensor can be attributed to the following factors:

Based on the above experimental results, F^−^ doping at 5 at% elevates the electron concentration of In_2_O_3_, thereby decreasing the baseline resistance (Figure 5d). Upon exposure to BTX gases, the released electrons from the surface oxidation reactions induce a more pronounced modulation of the EDL, producing a marked resistance variation and an enhanced sensing response.

The substitution of F^−^ for O_2_ in the In_2_O_3_ lattice produces $F_{O}^{+}$ (F^−^ occupying an O^2−^ site, with one positive effective charge) and one extra electron (e^−^). The released electron may subsequently be captured by $V_{O}^{+}$ (singly positively charged oxygen vacancy) or $V_{O}^{++}$ (doubly positively charged oxygen vacancy) to yield $V_{O}^{0}$ (neutral oxygen vacancy) or $V_{O}^{+}$, which effectively compensates the excess positive charge and ensures overall charge balance, as illustrated by Equations (8)–(10) [19].

(8) $$O_{O}\to F_{O}^{+}+e^{-}$$

(9) $$e^{-}+V_{O}^{++}\to V_{O}^{+}$$

(10) $$e^{-}+V_{O}^{+}\to V_{O}^{0}$$

Specifically, at low doping concentrations (0–5 at%), F^−^ ions mainly substitute for O^2−^ sites in the In_2_O_3_ lattice owing to their similar ionic radii. Each substitution donates one free electron to the conduction band, increasing the carrier concentration and decreasing the resistance. Beyond 5 at%, excess F atoms occupy interstitial rather than lattice sites. These interstitial atoms trap conduction electrons, inducing a self-compensation effect that reduces the effective carrier concentration. Simultaneously, enhanced ionized impurity scattering reduces carrier mobility, leading to a rebound in resistance. Obviously, the existence form of F exerts a substantial effect on the resistance regulation of In_2_O_3_ [49,50].

Furthermore, F incorporation induces lattice distortion and defect formation. The XRD results reveal lattice expansion of In_2_O_3_ after F doping, suggesting the generation of Ov. Consistently, the high-resolution O 1 s XPS analysis confirms increased O_V_ content from 35.8% for In_2_O_3_ to 40.6% for 5F-In_2_O_3_. Meanwhile, the O_C_ content increases from 26.4% for In_2_O_3_ to 34.3% for 5F-In_2_O_3_. The abundant defect sites promote O_2_ adsorption/activation and facilitate BTX oxidation, enhancing the response ability of the 5F-In_2_O_3_ sensor [28,51].

#### 3.3.2. Practical Application Evaluation

**Figure 10:**
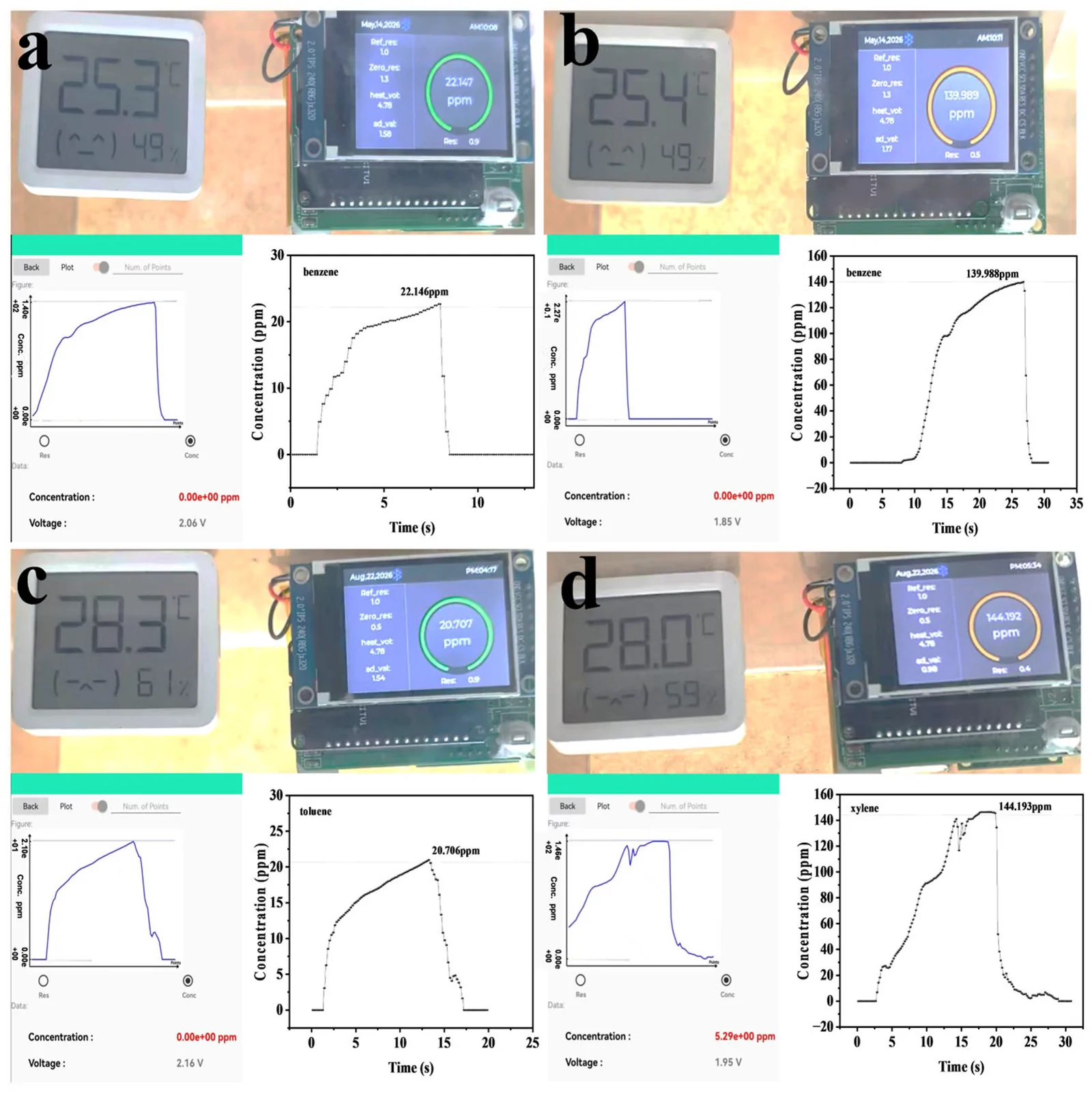
Photographs of the smartphone screen of the sensing software and the wireless sensing module, and the real-time gas sensing signal of the 5F-In_2_O_3_ sensor for (**a**) 20 ppm benzene, (**b**) 140 ppm benzene, (**c**) 20 ppm toluene and (**d**) 140 ppm xylene.

To evaluate the practical applicability of the 5F-In_2_O_3_ sensor for BTX monitoring, a portable wireless gas sensing module was fabricated (Figure 10 and Figure S6). Concentration gradient tests were conducted using a stepwise cumulative concentration method for benzene, toluene, and xylene. When the target concentrations of benzene, toluene, and xylene were increased to 20 ppm and 140 ppm, respectively, the measured concentrations obtained by the sensing module were 22.1 ppm and 139.9 ppm, 20.7 ppm and 138.3 ppm, and 19.7 ppm and 144.2 ppm, respectively. The measured values agreed well with the actual concentrations, confirming the module’s accurate quantitative detection capability. These results indicate the potential of the 5F-In_2_O_3_ sensor for practical BTX monitoring. However, due to inherent limitations in the current module, the prediction of gas concentrations in mixed BTX atmospheres was not achieved.

In this work, our primary goal is to evaluate the intrinsic sensing performance of F-doped In_2_O_3_ nanofibers toward BTX gases and to assess the feasibility of machine learning-assisted discrimination. The experimental design is mainly focused on the gas responses to individual BTX and the classification of binary BTX mixtures, which provides an initial validation of the effectiveness of the F-doping strategy and the capability of ML approaches for BTX discrimination. However, real application scenarios are far more complex than under laboratory conditions. In practical environments, multiple factors may affect sensor performance, including cross-interference from coexisting VOCs, baseline drift and sensitivity degradation due to surface poisoning. To address these challenges, we will extend our future work in several directions: (1) dynamic temperature modulation to obtain the specific transient fingerprints and improve selectivity, (2) enlarging the feature set (e.g., response slope or area under the curve) together with deep learning models for better multi-component recognition, (3) using the humidity compensation mechanism for real-time signal correction.

## 4. Conclusions

This study demonstrates an effective defect engineering strategy for enhancing the BTX sensing performance of In_2_O_3_ semiconductors. A series of F-doped In_2_O_3_ nanofibers were synthesized via an electrospinning method. The optimized 5F-In_2_O_3_ sensor exhibits superior BTX sensing performance compared with In_2_O_3_ at an operating temperature of 240 °C. Remarkably, the responses toward benzene, toluene, and xylene were enhanced by 4.3-, 4.5- and 4.2-fold, respectively. The limits of detection are reduced to 9.9 ppb, 5.7 ppb, and 2.5 ppb, respectively. Furthermore, extraction of characteristic features from dynamic response curves enables accurate discrimination of BTX and their binary mixtures, and a wireless sensing module was developed for BTX monitoring. Comprehensive characterizations revealed that F^−^ incorporation induced lattice distortion and abundant surface oxygen vacancies, facilitating oxygen adsorption/activation and accelerating BTX oxidation, thereby improving sensing performance. F doping has proven to be an effective strategy for developing high-performance and miniaturized BTX sensors.

## Figures and Tables

**Table 1:** Comparison of BTX gas sensing performance in reported gas sensors.

Sensing Materials	Operating Temperature (°C)	Gas/ Vapor	Con. (ppm)	Sensing Response	Res./Rec. Time (s)	LOD (ppm)	Ref.
WO_3_	300	Benzene	8	-	30/51	1.43	[40]
		Toluene		-	32/101	0.7	
		Xylene		-	43/108	0.3	
Au-ZnO	30 (UV light)	Benzene	1	27.9% (∆R)/R_a_ × 100%	35/137	0.1	[41]
		Toluene		39.3% (∆R)/R_a_ × 100%	40/133		
		Xylene		53.5% (∆R)/R_a_ × 100%	72/396		
Au NPs-ZnO	350	Benzene	25	30 (R_a_/R_g_)	134/512	<0.4	[42]
		Toluene		80 (R_a_/R_g_)	192/534		
		Xylene		120 (R_a_/R_g_)	258/546		
ZnO-CdO	room	Benzene	100	453 (R_a_/R_g_)	57/65	1	[43]
Ni-ZnO	325	Toluene	100	210.0 (R_a_/R_g_)	2/77	0.5	[44]
Mn-Co_3_O_4_	280	Toluene	100	91.2 (R_g_/R_a_)	55/30	5	[45]
Ir-c-WO_3_	180	Xylene	15	-	29/18	1	[46]
CoWO_4_/ Co_3_O_4_	200	Xylene	100	51.6 (R_g_/R_a_)	236/254	0.3	[47]
5F-In_2_O_3_	240	Benzene	50	217% (∆R)/R_g_ × 100%	3/6	0.0099	This work
		Toluene		394% (∆R)/R_g_ × 100%	2/9	0.0057	
		Xylene		514% (∆R)/R_g_ × 100%	1/12	0.0025	

Note: Con. denotes concentration, Res./Rec. time refers to response/recovery time, Ref. denotes reference. LOD refers to limit of detection.

**Table 2:** Average Accuracy of Eight Algorithms for Gas Identification.

Input Characteristics	Accuracy							
	RF	XGB	SVM	GB	KNN	MLP	LR	CNN
S, t_1_, t_2_	92.1%	86.8%	89.5%	81.6%	89.5%	86.8%	86.8%	86.8%

Note: RF: Random Forest; XGB: eXtreme Gradient Boosting; SVM: Support Vector Machine; GB: Gradient Boosting; KNN: K-Nearest Neighbors; MLP: Multilayer Perceptron; LR: Logistic Regression; CNN: Convolutional Neural Network.

## Data Availability

The original contributions presented in this study are included in the article and Supplementary Materials. Further inquiries can be directed to the corresponding authors.

## Supplementary Materials

The following supporting information can be downloaded at: https://www.mdpi.com/article/10.3390/nano16171119/s1, Figure S1. Dynamic responses of In_2_O_3_ sensor to various BTX concentrations at 240 °C: (a) benzene, (b) toluene, (c) xylene. Figure S2. Long stability over 15 days of 5F-In_2_O_3_ toward 50 ppm benzene, toluene, and xylene at 240 °C. (b) Response versus relative humidity (RH) for 5F-In_2_O_3_ toward 50 ppm benzene, toluene, and xylene at 240 °C. Figure S3. Linear fitting of response values of 5F-In_2_O_3_ toward 0.5–10 ppm benzene at 240 °C. Figure S4. Linear fitting of response values of 5F-In_2_O_3_ toward 0.25–10 ppm toluene at 240 °C. Figure S5. Linear fitting of response values of 5F-5F-In_2_O_3_ toward 0.05–10 ppm xylene at 240 °C. Figure S6. Photographs of the smartphone screen of the sensing software and the wireless sensing module, and the real-time gas sensing signal of the 5F-In_2_O_3_ sensor to (a) 140 ppm toluene and (b) 20 ppm xylene. Table S1. Fifty consecutive normalized baseline resistance data points measured in air atmosphere. References [52,53] are cited in the Supplementary Materials.
