# Supplementary material for: F-Doped In2O3 Nanofibers for Enhanced BTX Detection with a Portable Wireless Sensing Module
Source: Nanomaterials (Basel). 2026 Sep 6;16(17):1119. doi: 10.3390/nano16171119 (PMC13567400; doi:10.3390/nano16171119)
Supplement: Supplementary file 1 [file nanomaterials-16-01119-s001.zip › Final Supplementary Materials-NanomaterialsID-4509195.pdf]

# Supplementary Materials

# F-doped $In_2O_3$ nanofibers for enhanced BTX detection with a portable wireless sensing module

**Jiaqi Yang[1,2,3]*, Lisen Yuan[1,2,3], Yi Chen[1,2,3], Xiaobin Zhou[1,2,3], Xiaojuan Yan[1,2,3], Gang Zhao[2,3], Weiguang Ma[2,3]***

[1] College of Physical Electronics Engineering, Shanxi University, Taiyuan 030006, China； yuanlisen@sxu.edu.cn (L.Y.); 202422609002@email.sxu.edu.cn(Y.C.); xbzhou@sxu.edu.cn (X.Z.); yanxj@sxu.edu.cn (X.Y); gangzhao@sxu.edu.cn (G.Z.)

[2] State Key Laboratory of Quantum Optics Technologies and Devices, Institute of Laser Spectroscopy, Shanxi University, Taiyuan 030006, China Collaborative Innovation Center of Extreme Optics, Shanxi University, Taiyuan, 030006, China

[3] China Collaborative Innovation Center of Extreme Optics, Shanxi University, Taiyuan, 030006, China

* Correspondence: jqyang2023@sxu.edu.cn (J.Y.); mwg@sxu.edu.cn (W.M.)

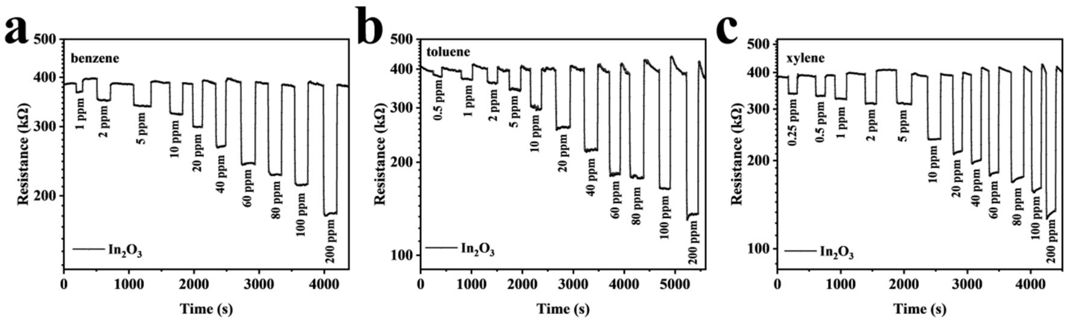


**Figure S1.** Dynamic responses of $In_2O_3$ sensor to various BTX concentrations at 240°C: (a) benzene, (b) toluene, (c) xylene.

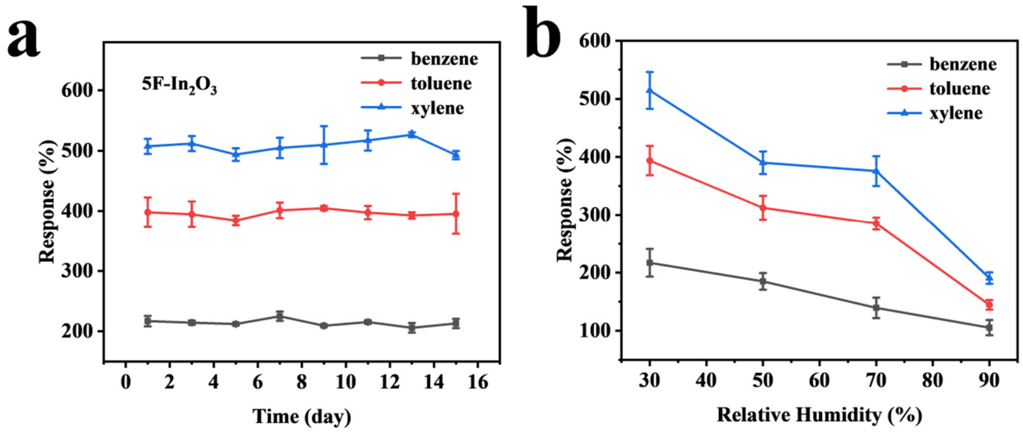


**Figure S2.** (a) Long stability over 15 days of 5F-$In_2O_3$ sensor toward 50 ppm benzene, toluene, and xylene at 240°C. (b) Response versus relative humidity (RH) for 5F-$In_2O_3$ sensor toward 50 ppm benzene, toluene, and xylene at 240°C.

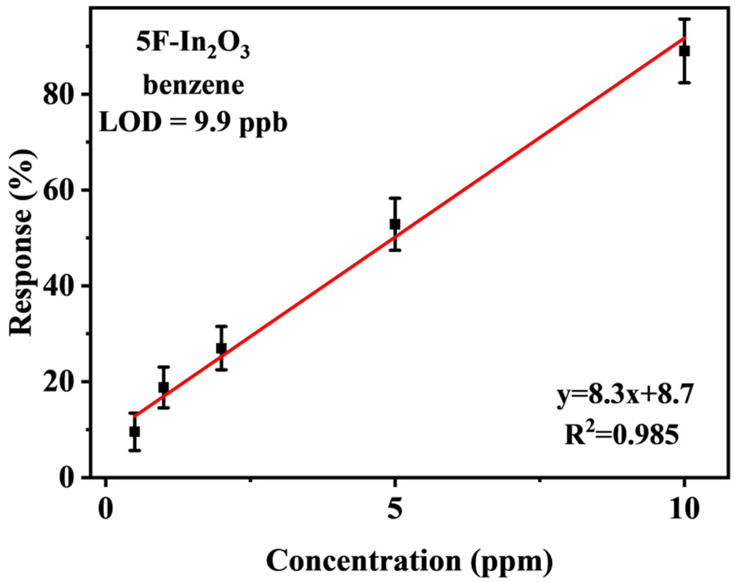


**Figure S3.** Linear fitting of response values of 5F-$In_2O_3$ sensor toward 0.5-10 ppm benzene at 240°C.

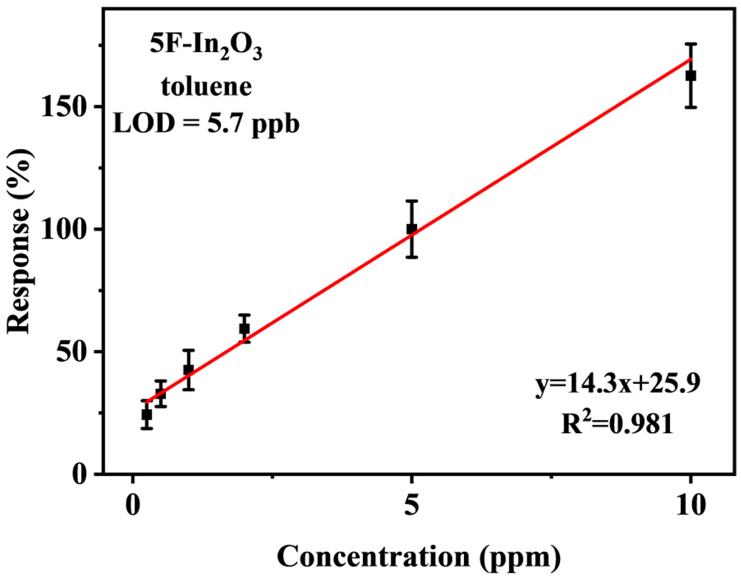


**Figure S4.** Linear fitting of response values of 5F-$In_2O_3$ toward 0.25-10 ppm toluene at 240°C.

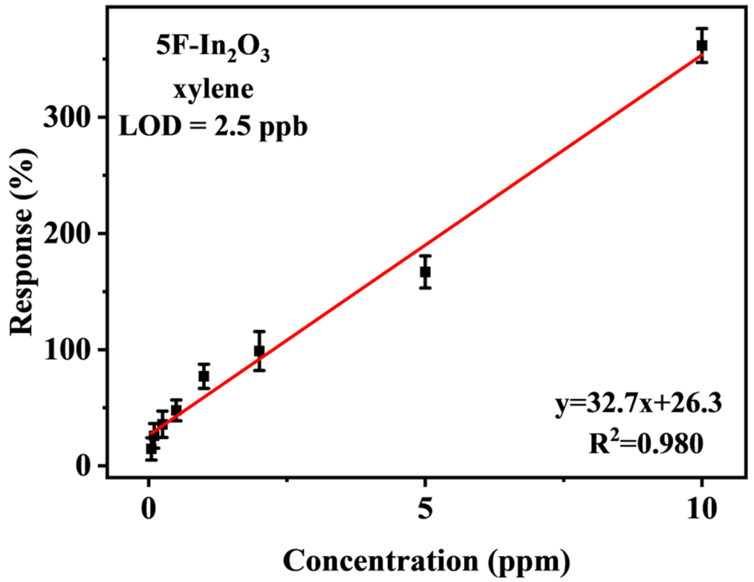


**Figure S5.** Linear fitting of response values of 5F-5F-$In_2O_3$ sensor toward 0.05-10 ppm xylene at 240 °C.

**Calculation method of the theoretical detection limit:**

$$RMS_{noise}=\sqrt{\frac{\sum(y_i-\bar{y})^2}{N}} \quad (1)$$

$$LOD=\frac{3\times RMS_{noise}}{Slope} \quad (2)$$

$RMS_{noise}$ is the root-mean-square (RMS) baseline noise, $y_i$ represents each normalized baseline resistance data point measured at the sensor baseline, $\bar{y}$ is the mean value of all selected normalized baseline resistance data points, slope is the sensitivity of the sensor obtained from linear regression of the response-concentration curve within the selected concentration range for each BTX analyte, and N is the total number of data points. The relevant data are presented in **Figure S3–S5** and **Table S1**.

**Table S1.** Fifty consecutive normalized baseline resistance data points measured in air atmosphere.

| | | | | | | | | | | |
|---|---|---|---|---|---|---|---|---|---|---|
| $y_i$ | 1.0117 | 1.0114 | 1.0108 | 1.0104 | 1.0098 | 1.0093 | 1.0088 | 1.0083 | 1.0078 | 1.0073 |
| | 1.0067 | 1.0061 | 1.0056 | 1.0051 | 1.0047 | 1.0042 | 1.0036 | 1.0031 | 1.0027 | 1.0023 |
| | 1.0020 | 1.0015 | 1.0011 | 1.0007 | 1.0002 | 0.9998 | 0.9993 | 0.9989 | 0.9985 | 0.9980 |
| | 0.9974 | 0.9969 | 0.9964 | 0.9959 | 0.99529 | 0.9948 | 0.99448 | 0.9939 | 0.99349 | 0.9931 |
| | 0.9927 | 0.9923 | 0.991 | 0.9916 | 0.9912 | 0.9907 | 0.9901 | 0.9899 | 0.9895 | 0.9890 |

Based on the normalized baseline resistance data listed in **Table S1**, the $RMS_{noise}$ was calculated to be 0.02733 using **Eq. S1**[1,2]**.** Taking benzene as an example, **Figure S3** shows the linear fitting of the response–concentration curve for benzene in the concentration range of 0.5-10 ppm. The linear relationship between the response of the 5F-$In_2O_3$ sensor and the benzene concentration was obtained as follows:

y=8.3x+8.7, Slope=8.3

Using **Eq. S2**[1,2], the theoretical detection limit for benzene was determined to be:

$LOD_{benzene}$=0.009878321 ppm≈9.9 ppb

By the same method, the theoretical detection limits for toluene and xylene were calculated to be approximately 5.7 ppb and 2.5 ppb, respectively.

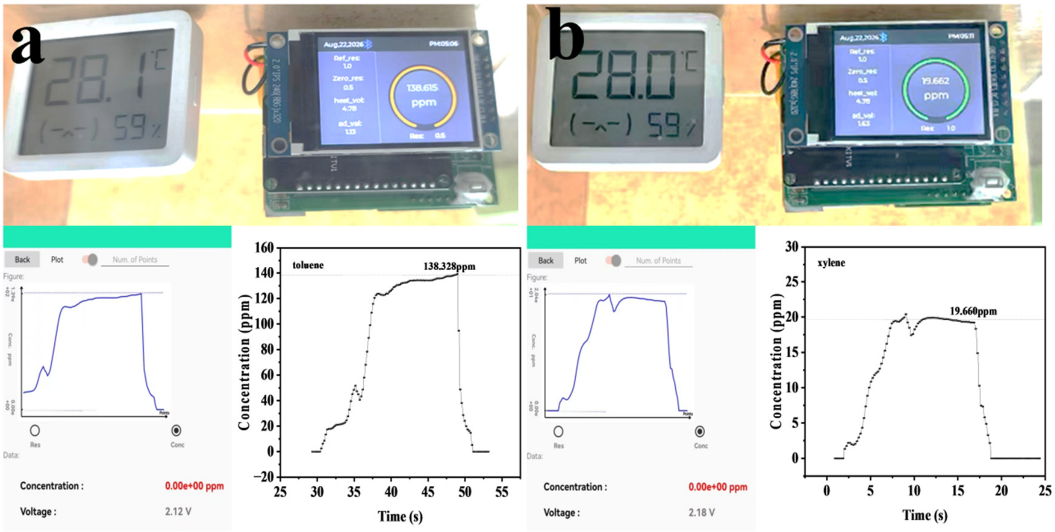


**Figure S6.** Photographs of the smartphone screen of the sensing software and the wireless sensing module, and the real-time gas sensing signal of the 5F-$In_2O_3$ sensor to (a) 140 ppm toluene and (b) 20 ppm xylene.

## References


1. Zhang, Y.; Jiao, J.; Han, W.; Zhang, H.; Shimanoe, K.; Zhou, T.; Sun, Y.; Lu, G. Synergistic carbon-doping strategy to simultaneously enhance sensing response and humidity resistance in $In_2O_3$-based $NO_2$ sensors. *Chemical Engineering Journal* **2026**, *527*, doi: 10.1016/j.cej.2025.171933.
2. Su, H.; Guan, Y.; Zhou, K.; Ma, C.; Hua, Z.; Wang, X.; Guo, X.; Zeng, D. Ultrasensitive response and ultralow detection limit of the acetone gas sensor based on 2D porous Pd-doped $WO_3$ nanosheets. *Ceramics International* **2025**, *51*, 25052-25062, doi: 10.1016/j.ceramint.2025.03.187.
